# Deep Learning Analysis of CT Images Reveals High-Grade Pathological Features to Predict Survival in Lung Adenocarcinoma

**DOI:** 10.3390/cancers13164077

**Published:** 2021-08-13

**Authors:** Yeonu Choi, Jaehong Aum, Se-Hoon Lee, Hong-Kwan Kim, Jhingook Kim, Seunghwan Shin, Ji Yun Jeong, Chan-Young Ock, Ho Yun Lee

**Affiliations:** 1 Department of Radiology, Sungkyunkwan University School of Medicine (SKKU-SOM), Samsung Medical Center, Seoul 06351, Korea; skyblue718@skku.edu; 2 Lunit Inc., Seoul 06241, Korea; brian.j.aum@lunit.io (J.A.); ssh@lunit.io (S.S.); 3 Division of Hemato-Oncology, Department of Medicine, Sungkyunkwan University School of Medicine (SKKU-SOM), Samsung Medical Center, Seoul 06351, Korea; shlee119@skku.edu; 4 Department of Thoracic Surgery, Sungkyunkwan University School of Medicine (SKKU-SOM), Samsung Medical Center, Seoul 06351, Korea; hkts@skku.edu (H.-K.K.); jkimsmc@skku.edu (J.K.); 5 Department of Pathology, Kyungpook National University School of Medicine, Kyungpook National University Chilgok Hospital, Daegu 41404, Korea; jyjeong@knu.ac.kr

**Keywords:** lung adenocarcinoma (ADC), heterogeneity, high-grade pattern, histology, prognosis, recurrence

## Abstract

**Simple Summary:**

The high-grade pattern (micropapillary or solid pattern, MPSol) in lung adenocarcinoma affects the patient’s poor prognosis. We aimed to develop a deep learning (DL) model for predicting any high-grade patterns in lung adenocarcinoma and to assess the prognostic performance of model in advanced lung cancer patients who underwent neoadjuvant of definitive concurrent chemoradiation therapy (CCRT). Our model considering both tumor and peri-tumoral area showed area under the curve value of 0.8. DL model worked well in independent validation set of advanced lung cancer, stratifying their survival significantly. The subgroup with a high probability of MPSol estimated by the DL model showed a 1.76-fold higher risk of death. Thus, our DL model can be useful in estimating high-grade histologic patterns in lung adenocarcinomas and predicting clinical outcomes of patients with advanced lung cancer who underwent neoadjuvant or definitive CCRT.

**Abstract:**

We aimed to develop a deep learning (DL) model for predicting high-grade patterns in lung adenocarcinomas (ADC) and to assess the prognostic performance of model in advanced lung cancer patients who underwent neoadjuvant or definitive concurrent chemoradiation therapy (CCRT). We included 275 patients with 290 early lung ADCs from an ongoing prospective clinical trial in the training dataset, which we split into internal–training and internal–validation datasets. We constructed a diagnostic DL model of high-grade patterns of lung ADC considering both morphologic view of the tumor and context view of the area surrounding the tumor (MC3DN; morphologic-view context-view 3D network). Validation was performed on an independent dataset of 417 patients with advanced non-small cell lung cancer who underwent neoadjuvant or definitive CCRT. The area under the curve value of the DL model was 0.8 for the prediction of high-grade histologic patterns such as micropapillary and solid patterns (MPSol). When our model was applied to the validation set, a high probability of MPSol was associated with worse overall survival (probability of MPSol >0.5 vs. <0.5; 5-year OS rate 56.1% vs. 70.7%), indicating that our model could predict the clinical outcomes of advanced lung cancer patients. The subgroup with a high probability of MPSol estimated by the DL model showed a 1.76-fold higher risk of death (HR 1.76, 95% CI 1.16–2.68). Our DL model can be useful in estimating high-grade histologic patterns in lung ADCs and predicting clinical outcomes of patients with advanced lung cancer who underwent neoadjuvant or definitive CCRT.

## 1. Introduction

Invasive lung adenocarcinoma (ADC) has been classified by the 2011 classification system of International Association for the Study of Lung Cancer (IASLC), American Thoracic Society (ATS), and European Respiratory Society (ERS) into five distinct histological patterns: lepidic, acinar, papillary, micropapillary, and solid [1]. Lung ADC is divided into low-, intermediate, and high-grade prognostic groups according to the most predominant pattern detected by histopathology [2,3,4]. However, even among lung ADCs with the same most predominant pattern, the spectrum of actual prognosis varies widely [3,5,6,7]. Moreover, regardless of the predominant pattern, the presence of any high-grade pattern such as a micropapillary and solid pattern is known to have a poor prognosis [6,8]. Therefore, identifying the presence of any high-grade pattern in lung ADCs before surgery can help predict a patient’s prognosis and determine a treatment policy. Especially in inoperable patients, which account for 80% of all lung cancer patients [9], it is difficult to determine the histologic pattern of the entire tumor with a very small biopsy sample. Therefore, there is a growing need for imaging biomarkers that can noninvasively predict high-grade patterns in lung ADCs.

In recent years, deep learning (DL) has become a powerful method of representation learning, decreasing the need for handcraft feature engineering [10]. DL technology is an artificial intelligence system based on neural networks that simulate the human brain using a simulation model called perceptron. Multi-layered perceptrons are constructed by creating and arranging layers of perceptrons in which all nodes of the model are fully connected in order to solve more complex problems.

Currently, DL is regarded as state of the art technology and is used to solve numerous problems in various fields [11]. In particular, in the field of biomedical science, the use of DL to identify an essential genes or create models that predict specific proteins offers the potential to replace cost- and time-consuming laboratory-based research [12,13]. Its effectiveness has been demonstrated in medical imaging analysis as well, such as detection of pulmonary nodules [14], classification of benign and malignant nodules [15], and prediction of tumor invasiveness [16,17]. However, few studies have used deep learning to classify histologic patterns in lung ADCs, which is difficult to perform with human eyes. Ding et al. trained two different deep learning models to predict a micropapillary pattern using LeNet and DenseNet, and showed an overall accuracy of 92 and 72.9% [18]. Wang et al. reported that combined radiomics and a deep learning model for the prediction of high-grade patterns in lung ADCs manifested as ground-glass opacity nodule (GGN) and resulted in an overall accuracy of 91.3% [19]. However, both studies are limited in that they only showed the technical success of the model. Therefore, our study attempted to evaluate not only the technical success of the model, but also how well the model can be applied in clinical situations.

The purpose of this study was to develop a deep learning model for predicting any high-grade pattern in lung ADCs and to assess the prognostic performance of this model in advanced lung cancer patients who underwent neoadjuvant concurrent chemoradiation therapy (CCRT) or definitive CCRT.

## 2. Methods

### 2.1. Study Design and Data Sources

Data from a prospective clinical trial of early-stage lung ADC patients who underwent preoperative contrast enhanced CT scans (NCT01482585) were used for the training dataset. The training dataset that was used to build the deep learning model consisted of 275 patients with 290 early lung ADCs recruited from Samsung Medical Center (SMC) between December 2011 and January 2017. Some patients had synchronous lung cancer, so the number of lesions was greater than the number of patients. The inclusion criteria were clinical stage I or II lung ADC with a performance status of 0 to 1 on the ECOG scale and age of 20 years or older. Patients with a history of previous radiation or chemotherapy and poor cardiopulmonary reserve were excluded.

**Figure 1:**
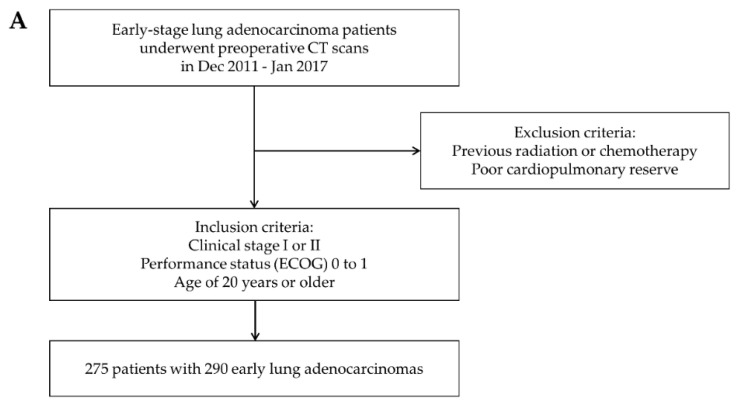

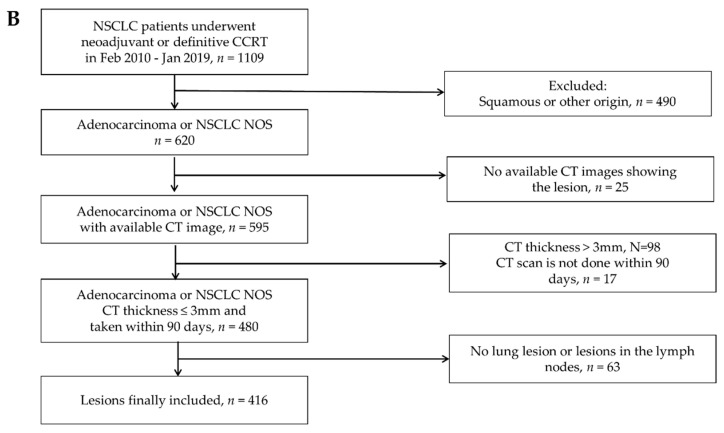
Patient inclusion process. (**A**) Inclusion criteria for the training data set. (**B**) Inclusion criteria for the validation data set.

For the validation dataset, we retrospectively evaluated an independent dataset of 416 patients with advanced non-small cell lung cancer (NSCLC) who underwent neoadjuvant concurrent chemoradiation therapy (CCRT) or definitive CCRT at SMC between February 2010 and January 2019. We included patients with stage I A to III B lung ADC who had matched contrast-enhanced CT images performed within 90 days before and after biopsy. Only CT images using thicknesses within 3 mm were included. Patients who were diagnosed with NSCLC of a cell type other than ADC or had CT images that did not meet the above criteria were excluded. Cases in which primary tumors were not clearly seen in the lung or only lymph node metastases were observed were also excluded. Finally, 416 patients were included in the validation dataset. All these patients’ inclusion processes are illustrated in Figure 1A,B. All patients provided written informed consent, and study approval was obtained from the Institutional Review Board of SMC, Seoul, Korea.

Any high-grade pattern inside the tumor predicted by the DL model was denoted as MPSol. MPSol is defined as the presence of more than 5% micropapillary or solid histologic patterns inside the tumor.

Overall survival (OS) was calculated from the date of the surgery or radiation therapy to that of death from any cause. Disease-free survival (DFS) was calculated from the date of surgery to that of tumor recurrence or the data of the last follow-up. Progression-free survival (PFS) was calculated from the date of radiation therapy to that of tumor progression or the date of the last follow-up.

### 2.2. Preprocessing

The CT scans were obtained from the SMC. The scanning matrix was set to 512 × 512 pixels. For training cohort, the slice thickness was 1.0 mm. For the validation cohort, it ranged from 1 to 3 mm. 

AVIEW Corelinesoft software (Coreline Soft, Seoul, Korea) was used to label the tumors in the images. The target lesion was marked by an experienced radiologist (5 years of experience in chest CT diagnosis) as 3D segmentation mask. Then, a cuboid bounding box that tightly covered the target lesion was created.

There were preprocessing steps of image as follows. Every 2D slices of DICOM images were stacked to create 3D pixel matrix with Hounsfield Units (HU) as pixel value. The 3D pixel matrix was then resampled to have 1.5 mm × 1 mm × 1 mm (depth × width × height) resolution with linear interpolation. Pixel values were clipped to have both the lung window (−1400 HU, 200 HU) and the mediastinal window (−125 HU, 225 HU) and then rescaled to between 0 and 1. Both views are concatenated channel-wise before being fed into the deep learning model, allowing the model to observe both the lung window and the mediastinal window as a human radiologist does.

**Figure 2:**
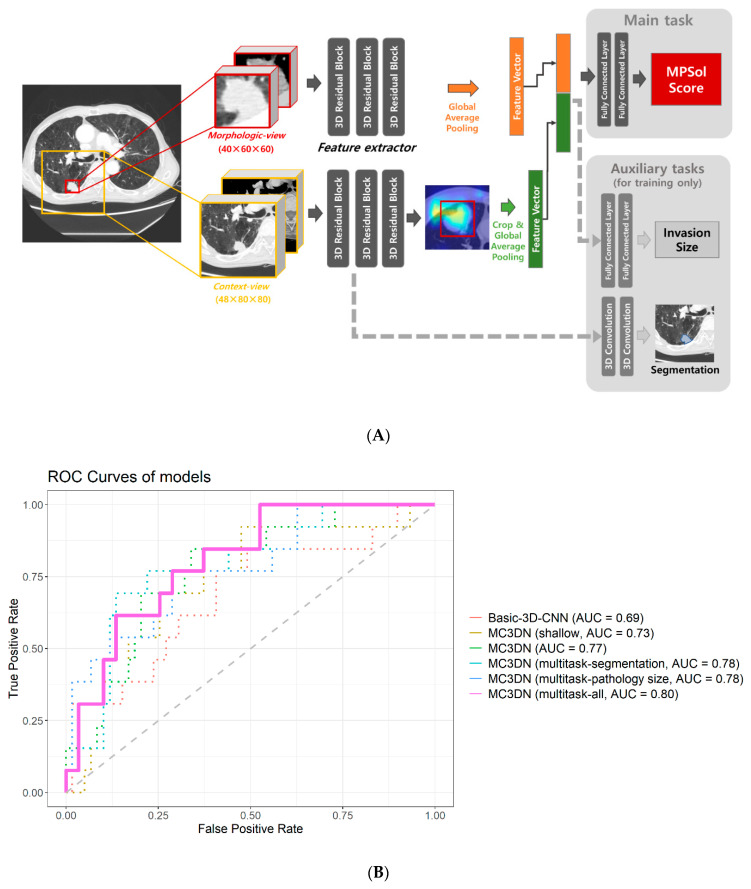
(**A**) Deep learning workflow. (**B**) Performance of the model. ROC curves generated from different models to predict any high-grade pattern of lung ADCs. The basic-3D-CNN model takes only one 3D patch from target lesion with a model architecture consisting of one residual block for each level (AUC = 0.69). The Morphologic-view Context-view 3D network (MC3DN) is introduced. The MC3DN (shallow) model takes both the context and morphologic views from the target lesion while having the same model architecture as Basic-3D-CNN (AUC = 0.73). A larger model architecture is adopted for MC3DN with three residual blocks for each level (AUC = 0.77). A multitasking strategy is applied with the segmentation task and pathology size prediction task, respectively, on MC3DN (AUC = 0.78). Finally, both of the tasks are applied on MC3DN simultaneously (AUC = 0.8).

Two types of 3D patches were extracted from the target lesion. One is what we call the context-view, which is cropped from a 144 mm × 160 mm × 160 mm (depth × width × height) region around the target lesion and resampled to have 48 × 80 × 80 pixels containing a broad background area around the lesion. The other is the morphologic-view, which is tightly cropped as cuboid patch from the target lesion, then resampled to have 40 × 60 × 60 pixels. The detailed procedure is shown in Figure 2A.

Unlike the conventional radiomics research, we did not apply any feature extraction steps such as binning, intensity histogram (IH), gray-level co-occurrence matrix (GLCM) to let our deep learning model to learn to extract relevant features by itself.

When training our model, we applied multiple augmentation techniques to increase amount of data by adding slight modification on images [20]. Specifically, brightness and contrast jittering, resizing, bounding box jittering were applied on images every time by modifying 5% before feeding the images into model. For example, contrast jittering was applied by multiplying the number drawn randomly between 0.95 and 1.05 on pixel values of image with different random number for every iteration. Moreover, random flipping was also applied in axial, horizontal, and vertical axis by 50% of probability, respectively.

### 2.3. Model Architecture

#### Model: MC3DN

Our model was developed to focus on the morphologic characteristics of the target lesion as well as contextual information observed around the lesion (Figure 2A). We call our model the morphologic-view context-view 3D network (MC3DN). Two feature extractors are separately assigned to the context view and the morphologic view. The 3D convolutional model showed great success in the video classification task [21]. We employed it for our feature extractor which has some modifications as follows. It consisted of one convolution layer which takes a 3D image and the following three levels of three residual blocks. Each residual block consists of two 3D convolution layers. Two down sampling layers are located at the end of the first and the second levels, respectively. This results in 19 3D convolution layers in total, which is a very deep 3D neural network. The feature vectors produced from each feature extractor are concatenated in channels and fed into a classifier layer to predict a probability score between 0 and 1 for each lesion that indicates whether the target lesion is MPSol or not. Before we fed the feature vectors into the classifier layer, we applied global average pooling on the both the context view and the morphologic view to produce feature vectors. For the context-view, we cropped the feature vector around the target lesion as given by the annotation in the form of bounding box to separate the target lesion from other lesions which may present in the context view due to its broad field of view. Hyperparameters for the model were searched such as learning rate, learning schedule, network size, level of augmentation, etc. by conducting multiple experiments to find the best performance model.

### 2.4. Multitask Learning

Joint training of a main task with auxiliary tasks can be a very effective method to improve performance in the main task, especially when only small number of training samples are present [16,22]. Two additional auxiliary tasks are included in our training process. One is pathologic invasion size prediction and the other is a lesion segmentation task. Invasion size was estimated from pathology images by a pathologist. We assigned an additional classifier which takes the final feature vector produced from feature extractors to predict invasion size. For the segmentation task, two additional 3D convolutional layers are branched out from the initial residual block. The final segmentation mask was half the size of the context view. Our loss functions were given as cross-entropy loss after soft-max function for MPSol and segmentation mask prediction and smoothed-L1-loss for pathologic invasion size prediction. As the auxiliary tasks only help our main task (MPSol prediction), tasks are weighted 10:1:1 (MPSol prediction: pathologic invasion size prediction: lesion segmentation).

The research was performed with 8 Nvidia Titan xp graphics processing units (GPU). Our models were developed with Python 3.7 and PyTorch 1.0.1 on an Ubuntu 16.04 platform. 

### 2.5. Activation Mapping of the Deep Learning Model

To investigate whether the DL model is capable of understanding histologic patterns from CT images, we visualized the region that the model considered important on CT images for the given lesion and compared this with histopathologic images. In order to visualize where the model considered important inside the lesion, we visualized the activation maps of final convolution layer from the morphologic view. More specifically, we calculated the magnitudes of gradients flowing through the final convolution layer and merged the activation maps by weighted-averaging these with the magnitudes of gradients to highlight the most influenced activation maps on the final prediction [23].

### 2.6. Statistical Analysis 

A 2-tailed *p* value less than 0.05 was considered to be statistically significant. Receiver operating characteristic (ROC) curves were applied to evaluate the two-class classification models. Area under the curve (AUC) was calculated from ROC curves with a 95% confidence interval (CI). The Kaplan–Meier method was used for survival analysis and the log-rank test was used for statistical testing of survival performance. Hazard ratios and 95% CIs were computed using the Cox proportional hazards model. All statistical analyses were carried out using R 3.6.1 software (Vienna, Austria; http://www.R-project.org/). 

## 3. Results

### 3.1. Dataset Characteristics

Table S1 describes the clinicopathologic characteristics of patients in the training dataset. In terms of histologic grading of the most predominant pattern, 48 (16.6%) tumors were low-grade, 214 (73.8%) were intermediate grade, and 28 (9.6%) were high-grade. Of the 290 lung ADCs, 54 (18.6%) lesions showed any high-grade pattern (micropapillary or solid pattern, MPSol) in >5% of the pathological specimen. Of the 275 patients in the training set, 31 (11.3%) experienced recurrence. The mean DFS was 49.9 months, and the median follow-up period was 1763 days (range: 29–3228 days). Eleven patients died during follow-up, and six of these patients died from recurrent lung cancer. 

The validation dataset included 416 patients with advanced lung ADCs who underwent neoadjuvant or definitive CCRT (Table S2). Of the 416 patients in the validation set, 88 expired for any cause. The mean OS was 26.9 months, and the median follow-up period was 806 days (range: 7–3550 days).

### 3.2. Survival by High-Grade Pattern in Training Set

Survival curves for OS estimated by the Kaplan–Meier method differed significantly depending on the presence of any high-grade histologic pattern in lung ADCs (Figure S1, *p* = 0.005). The 5-year OS rate of any high-grade pattern was 88.2%, compared to low-intermediate grade (96.4%). There was also a significant difference in DFS (Figure S2, *p* < 0.001); the 5-year disease-free survival rate of any high-grade pattern was 77.8%, which was lower than that of the low-intermediate grade (87.3%).

### 3.3. Model Performance

The training cohort is split into internal–training and internal–validation datasets in a 3:1 ratio (*n* = 218:*n* = 72). We trained our model using an internal-training dataset to predict MPSol, and its performance was measured using ROC AUC for every epoch using the internal–validation dataset. We picked the best performance model from the internal-validation dataset to avoid overfitting on the internal–training dataset.

Initially, we developed a very basic 3D convolutional residual architecture model with one residual block for each level, which takes a 1.5 mm × 1 mm × 1 mm resampled CT image with 48 × 80 × 80 sized 3D patch of target lesion. This had an AUC of 0.6871 (95% CI 0.5146–0.8596). When we applied our MC3DN with one residual block for each level, the AUC value improved to 0.7301 (95% CI 0.5778–0.8825). As we increased the model size to three residual blocks for each level, there was an additional performance boost to an AUC of 0.7692 (95% CI 0.6359–0.9025). The performance of the baseline model from training cohort was measured 0.72 in ROC AUC with lung window only, while it was measured to be 0.78 when both lung and mediastinal windows are used. Multi-task learning also allowed performance gains. When the pathologic size was considered together, the AUC value was 0.7823 (95% CI 0.6442–0.9204). When segmentation was considered together, the AUC value was 0.7784 (95% CI 0.6352–0.9215). The AUC value was 0.8044 (95% CI 0.6861–0.9228) when considering pathologic size and segmentation, which was highest among several models. AUC of all models showed significant predictive values, but there was no significant difference between each model. The performances of all the models were measured from the internal–validation dataset and are shown in Figure 2B.

### 3.4. Validation of the Deep Learning Model

Univariable and multivariable Cox regression analysis were performed to determine the prognostic factors that contribute to patient survival in the validation set (Table 1). After adjusting forsex, stratified multivariable Cox analysis was performed on variable that did not meet the proportional hazards assumption (e.g., smoking). Moreover, the MPSol probability score was significantly associated with overall survival (HR 1.622, 95% CI 1.058–2.488, *p* = 0.027). There was no significant difference of survival between neoadjuvant and definitive CCRT groups (*p* = 0.950).

**Figure 3:**
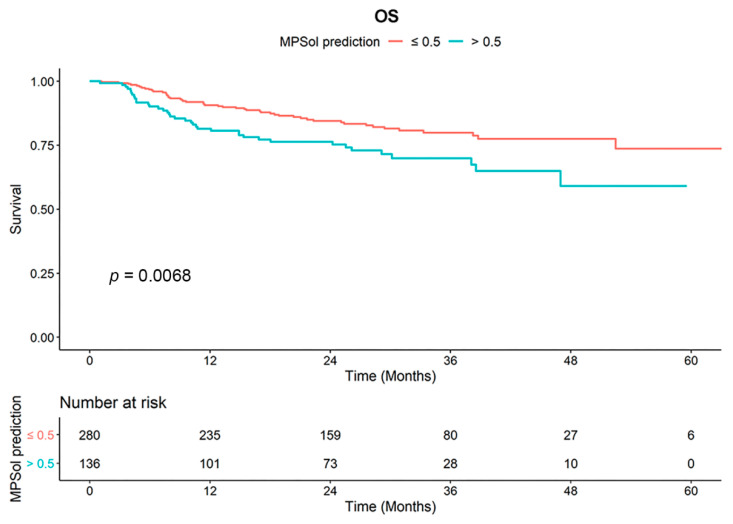
Survival curves for OS according to MPSol probability estimated by MC3DN in the validation set. When the deep learning model was applied to the validation set, a high probability of a high-grade histologic pattern such as the micropapillary and solid pattern (MPSol) was associated with worse overall survival (probability of MPSol >0.5 vs. <0.5; 5-year OS rate 59.0% vs. 73.6%).

Survival curves for OS and PFS stratified by the probability of a high-grade pattern estimated by MC3DN were significantly different (Figure 3 and Figure S3). The 5-year overall survival rate was 73.6% in the subgroup (MPSol probability < 0.5, *n* = 280) where the probability of the presence of a high-grade histologic pattern was less than 0.5. On the other hand, the 5-year survival rate was as low as 58.6% in the subgroup (MPSol probability > 0.5, *n* = 136) where the probability of the presence of a high-grade pattern was greater than 0.5. The subgroup with MPSol probability > 0.5 estimated by MC3DN showed a 1.78-fold higher risk of death (95% CI 1.17–2.72, *p* = 0.008) than the subgroup with MPSol probability < 0.5. Survival curves for all patients and other clinical factors are shown in Figure S4. In this validation dataset, only age showed a significant difference in overall survival. In the same analysis for PFS, a more favorable outcome was shown in the subgroup with MPSol probability > 0.5, but there was no significant difference (Table S3 and Figure S1). Exploratory subgroup analysis showed that the prognostic impact of MPSol probability was clearly significant in the patients with neoadjuvant CCRT treatment (*n* = 218, *p* = 0.00085), but it was not significant in those with definitive CCRT treatment (*n* = 198, *p* = 0.51) (Figure S5). Along with this result, we observed that the proportions of N stage of two groups (neoadjuvant versus definitive CCRT) were different (0.9% versus 66.7%, respectively), implying that N3 node would confer a distinct CT image pattern compared to its primary lesion.

### 3.5. Activation Mapping of the Deep Learning Model

**Figure 4:**
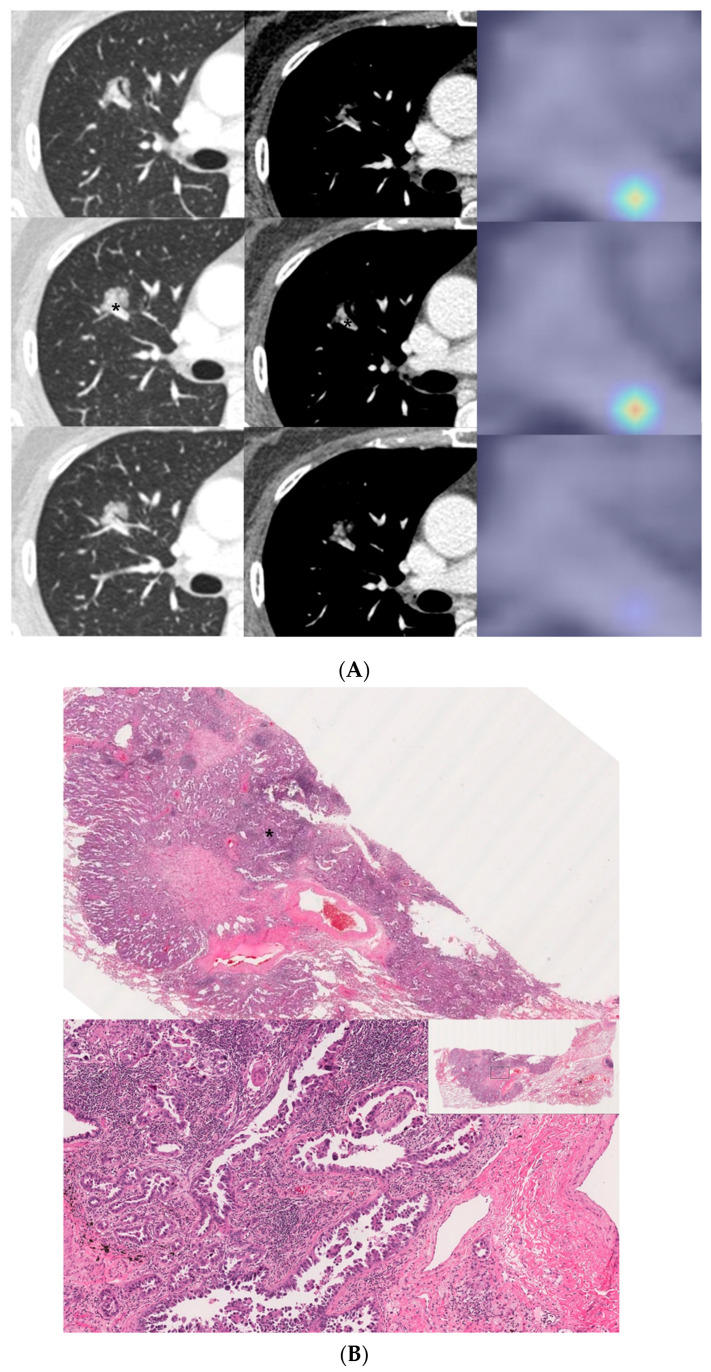
A 57-year-old female with invasive lung adenocarcinoma (Acinar 90%, MP 1%, Scar 9%). (**A**) A 13 mm part-solid nodule with air-bronchograms is noted in right upper lobe. The activation map is shown as a heatmap, highlighting the most important region when it classified the given lesion as MPSol (micropapillary or solid pattern). A very focal highlighted region (marked with an asterisk) is noted in the proximal portion of the tumor, adjacent to a transversing vascular structure. (**B**) Surgical pathology demonstrates a micropapillary histologic pattern (marked with an asterisk) adjacent to the vascular structure in the tumor which corresponds to the activation map.

**Figure 5:**
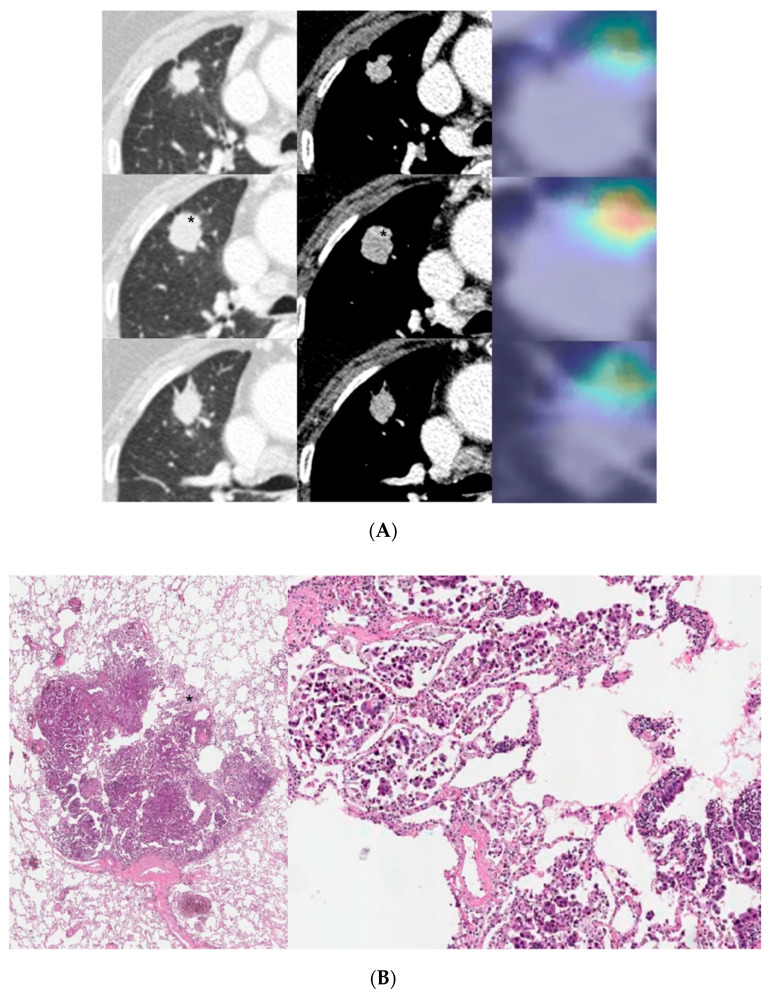
An 81-year-old male with invasive lung adenocarcinoma (Acinar 88.3%, MP 5.6%, Scar 6.1%). (**A**) A 21 mm lobulated enhancing nodule is noted in the right upper lobe. The activation map is shown as a heatmap, highlighting the most important region (marked with an asterisk) when it classified the given lesion as MPSol (micropapillary or solid pattern). The focal highlighted region is noted on the anteromedial side of the tumor. (**B**) Surgical pathology demonstrates a focal micropapillary pattern (marked with an asterisk) on the anteromedial side of the tumor with background of an acinar pattern, which corresponds to the activation map.

The activation map visually presents the most important region that the deep learning model focused on when predicting an outcome. When we compared the activation map between the CT image and histology image, we were able to observe a correlation between the model-highlighted region and the corresponding region in the histology images. For example, when our model predicted MPSol, the region that the model highlighted correlated well with the micropapillary or solid region from histology images, as shown in Figure 4 and Figure 5, and Figures S6–S8.

## 4. Discussion

Lung ADCs are characterized by a heterogeneous mixture of histologic patterns of prognostic significance. Although the most predominant histologic pattern is known to be important for patient prognosis, the presence of any high-grade pattern such as micropapillary and solid patterns also has a significant impact on prognosis [2,6]. According to a paper recently published by the IASLC pathology group, the predominant plus high-grade pattern classified patient prognosis better than the predominant pattern alone [24]. Thus, the current study developed a deep learning model based on 3D convolutional neural networks and multitask learning, which automatically predicts any high-grade histologic pattern from CT scans of early-stage lung ADCs. We found that our deep learning model could be used to predict clinical outcomes in patients with advanced lung cancer who underwent neo-adjuvant or definitive CCRT.

In our study, patients with early-stage lung cancer had a significantly poorer prognosis when there was any high-grade histologic pattern (Figure S1). These results are consistent with previous studies [2,6,7] and reaffirmed the importance of predicting any high-grade histologic pattern. However, although we know that the prognosis is worse with the high-grade pattern, it is difficult to know in advance the existence of a high-grade pattern before surgery. In addition, surgical decision-making based on frozen sections of high-grade histologic patterns has little evidence to prove diagnostic accuracy, which can be problematic when considering sub-lobar resection [25]. In this respect, our DL model is meaningful because it noninvasively predicts high-grade histologic patterns using CT scans prior to obtaining pathologic specimens. Tsao et al. reported that patients with micropapillary and solid patterns could benefit from adjuvant chemotherapy and had an improved disease-free survival rate [26]. As such, using our DL model could help clinicians determine treatment options such as adjuvant chemotherapy or determining an appropriate resection margin.

There have been many studies on the imaging-based deep learning model for predicting the histology of lung cancer (i.e., adenocarcinoma, squamous cell carcinoma, and small cell lung cancer), and it has shown relatively good accuracy [27,28,29,30]. However, there are few studies on radiomics-based machine learning or CT-based deep learning models predicting high grade histologic patterns of lung ADC (i.e., micropapillary or solid pattern, MPSol). He et al. developed a radiomics-based machine learning model to predict MPSol for patients with curative invasive lung ADC and showed good predictive performance (AUC, 0.75) [31]. Wang et al. created both radiomics-based machine learning and deep learning-based models for predicting MPSol in patients with early-stage lung ADC presented as GGOs [19]. They found that the AUC values of each model were as follows: radiomics-based model, 0.673–0.819; deep learning-based model, 0.689; model combining deep learning and radiomics (DRL), 0.861. They reported that the model combining deep learning and radiomics performed the best, and in fact, their deep learning model had relatively low performance with AUC 0.689. The radiomics-based model presented in both studies showed good predictive performance similar to ours, but their study is different from deep learning-based research in that they used radiomics that had to go through the process of handcraft feature extraction. In addition, while Wang et al. built a model only for lesions represented by GGOs, we developed a model using all lesions that could be encountered in real clinical world, from GGO to solid lesions. As a study that built a deep learning-based model similar to our study, Ding et al. developed two different deep learning models, based on the LeNet and the DenseNet architecture for invasiveness classification and prediction of micropapillary pattern (MP) within lung ADC. They reported that the accuracies of each model for MP prediction were 92% and 73%, respectively [18]. However, there are limitations in that only the MP pattern was predicted, excluding the solid pattern, and the model was not validated in other cohorts. To the best of our knowledge, our model is the first to predict micropapillary or solid histologic patterns in a variety of tumors from early to advanced stages with comparable predictive performance.

The strength of our study is that we validated the DL model in patients with advanced lung cancer. It is difficult to prognosticate in patients with advanced lung cancer undergoing neoadjuvant or definitive CCRT with known histologic patterns because tissue information is not available. In addition, even if tissue information is obtained, there is the problem of obtaining only a small amount of biopsy sample that does not reflect the entire tumor. Therefore, there is an increasing need to stratify prognosis noninvasively by imaging, especially in patients with advanced lung cancer. When our DL model was validated for patients with advanced lung cancer without tissue information, it was confirmed that prognostic performance worked quite well. This is meaningful in that the DL model that predicts any high-grade histologic pattern in early lung cancer patients is well applied to advanced lung cancer. In addition, this suggests that a good grasp of the specific radiologic clues for the presence of a small proportion of high-grade patterns can impact more accurate prognostication.

Another strength of our study is that the activation map of our DL model has the potential to approximately correlate with the patient’s CT and histologic images.

Interestingly, in the cases of a small MPSol as shown in Figure 4 and Figure 5 and Figure S6, the focal area highlighted in the activation map was actually correlated with the focal area of micropapillary or solid pattern in the pathology. In addition, in cases with high MPSol, as shown in Figures S7 and S8, the entire tumor area highlighted in the activation map was correlated with a diffuse area of micropapillary or solid pattern in the pathologic images. This suggested our model has the power to discriminate heterogeneous histologic patterns for given lesions from CT images only.

There are several factors that explain the performance of our deep learning model. First, our model considered both morphologic and context views, which means that our model considered not only the tumor, but the surrounding environment of the tumor as well. Similarly, Hosney et al. reported that tissues both within and beyond the tumor were crucial for characterization and eventual prediction in a DL model for lung cancer prognostication [32]. Interestingly, our model used a relatively wide context view (144 mm × 160 mm × 160 mm). Thus, the performance of our model is interpreted as the result of reflecting a wide area of lung parenchyma around the tumor as well as the imaging properties of the tumor itself. Second, the performance improved by 2.8% when the model was trained with both lung and mediastinal windows on CT scans. Compared to the lung window, the mediastinal window makes the tumor appear smaller and has a simpler border. In other words, considering that both windows may explain the characteristics of the margin of the tumor, this can provide richer information to the model. Third, pathologic invasive size was also trained through multitask learning and contributed to the improvement of model performance. As revealed through many studies [33], pathologic invasive size is one of the most important factors in determining patient prognosis. Through joint training of the neural networks to solve two related tasks, the model is able to predict a patient’s prognosis more accurately. This multitask learning approach is also effective at learning convergence and the system is less prone to overfitting. Thus, by considering pathologic invasive size as well as histologic pattern, we were able to create a more robust model for predicting patient prognosis.

There are no reports of performance comparisons between radiologists and deep learning models for predicting high-grade histologic patterns such as micropapillary or solid. This is because it is difficult to predict a high-grade pattern by simple visual assessment, because even the same solid tumor may or may not have a high-grade pattern inside. Moreover, especially when the high-grade pattern is a non-predominant pattern, it will be difficult to expect good performance from visual assessment alone. This reinforces the need for our deep learning model in that our model can predict what is difficult for a radiologist to predict under usual circumstances.

Several limitations are left to be addressed in our study. First, data insufficiency in the training session could lead to bias. To overcome this problem, we applied various image augmentation techniques and implemented multitask learning. In addition, Song et al. combined imaging with clinical features to identify pathologic components [34], by training imaging parameters as well as the relevant clinical information; we could provide richer information to the model and create a more robust model. Second, we did not perform external validation with a similar population. Instead, we validated the model in a completely different cohort of advanced lung cancer patients treated by neoadjuvant or definitive CCRT. Although the prognostic impact of MPSol probability is clearly different according to treatment groups, possibly caused by the imbalanced proportion of N3 stage, we also verified the model’s performance by showing that it predicts prognosis well in the merged group. Third, there was a difference in the range of tumor sizes between the training and validation sets. In other words, the validation set consisting of advanced lung cancer patients had an overall larger tumor size than seen in the training set. However, there was no extreme outlier in the size distribution of each groups. The longest CT diameters of the tumors ranged from 5.5 to 74.1 mm in training set, and 8.9 to 127.6 mm in validation set. The median and interquartile ranges of tumor size were as follows for each dataset: median [quartile 1, quartile 3], 22.1 mm [16.6, 31.5] in training set; 36.8 mm [25.7, 52.5] in validation set. Although the longest diameter of the tumor was naturally larger in the validation set due to the nature of advanced lung cancer, it is rather meaningful that our model nevertheless worked well. Fourth, although our model was developed through training on lung ADCs, it was validated in patients diagnosed with NSCLC NOS as well as ADCs irrespective of histologic subtype. However, this is a plausible approach as patients have various histologic subtypes other than ADCs in real clinical settings. Moreover, as our model actually worked well in the validation set with a mixture of different histologic subtypes, it will be easier for clinicians to apply to real-world clinical situations. Fifth, our study is a single-center study that can be biased. Further validation may be required in a larger cohort drawn from other institutions. Last, DL systems have an inherent problem with interpretability. Compared to engineered radiomics [35], feature definition, extraction, and selection in deep learning are all automated by black-box-like networks. Thus, it is difficult to debug these opaque networks, isolate the cause of certain outcomes, and predict when and where failures will occur.

DL has shown great potential in fields with imaging data such as radiology [36], pathology [37], dermatology [38], and ophthalmology [39]. In our study, we also used CT images to build a DL model, which was quite useful in predicting a patient’s prognosis through high-grade histologic patterns. In addition to DL application in imaging classification, DL has a multimodal nature that can integrate various fields such as genomic, pathologic, and clinical information. Therefore, by incorporating genetic and clinical information into our DL model, we could build a better and more robust model in the future. Our DL model may be able to contribute to precision medicine by enabling patient-specific diagnosis and treatment.

## 5. Conclusions

In conclusion, our deep learning model could be useful in estimating high-grade histologic patterns in lung ADCs and predicting clinical outcomes of patients with advanced lung cancer who underwent neoadjuvant or definitive CCRT. Our model is meaningful in that it has been successfully applied to inoperable patients and could help to determine the treatment policy for these patients.

## Figures and Tables

**Table 1:** Cox Regression Analysis for Overall Survival in Validation Set.

	Univariable		Multivariable ^#^	
	HR (95% CI)	*p* Value	HR (95% CI)	*p* Value
Treatment: Neoadjuvant	1.014 (0.666–1.544)	0.950	-	-
Sex: male	2.217 (1.333–3.688)	0.002 *	1.453 (0.709–2.978)	0.308
Age	1.013 (0.990–1.037)	0.256	-	-
Smoking: yes	2.123 (1.354–3.330)	0.001 *		
ECOG (≥1)	0.858 (0.492–1.498)	0.591	-	-
MPSol prediction >0.5	1.781 (1.166–2.721)	0.008 *	1.622 (1.058–2.488)	0.027 *
TNM stage: I, II (Ref)				
TNM stage: III	1.083 (0.472–2.483)	0.851	-	-

* *p* < 0.05, ^#^ Stratified by smoking, Smoking status included both former and current smokers.

## Data Availability

Data are available in a publicly accessible repository.

## Supplementary Materials

The following are available online at https://www.mdpi.com/article/10.3390/cancers13164077/s1, Figure S1: Survival curves for OS according to any high-grade histologic pattern of lung ADCs in training set. Overall survival was significantly different between lung ADCs with or without any high-grade histologic pattern such as micropapillary and solid pattern (*p* = 0.005, 5-year OS rate; 96.4% vs. 88.2%), Figure S2: Survival curves for DFS according to any high-grade histologic pattern of lung ADCs in training set. DFS was significantly different between lung ADCs with or without any high-grade histologic pattern such as micropapillary and solid pattern (*p* < 0.001, 5-year DFS rate; 87.3% vs. 77.8%), Figure S3: Survival curves for PFS according to MPSol probability estimated by MC3DN in the validation set. When the deep learning model was applied to the validation set, a high probability of a high-grade histologic pattern such as the micropapillary and solid pattern (MPSol) was associated with worse progression free survival (probability of MPSol >0.5 vs. <0.5; 5-year PFS rate 47.0% vs. 36.5%), Figure S4: Survival curves for all patients in validation cohort and other clinical factors, Figure S5: Survival curves for OS according to MPSol probability estimated by MC3DN in neoadjuvant or definitive CCRT group, Figure S6: A 71-year-old male with invasive lung adenocarcinoma (Papillary 65%, Solid 30%). (*A*) A 30 mm sized lobulated heterogeneously enhancing mass is noted in right upper lobe. Activation map visually as heatmap, highlighting the most important region (marked with an asterisk) when it classified the given lesion as MPSol (micropapillary or solid pattern). A focal highlighted region is noted in central portion of the tumor. (*B*) Surgical pathology demonstrates solid histologic pattern (marked with an asterisk) in central portion of the tumor which corresponds to activation map, Figure S7: A 60-year-old male with invasive lung adenocarcinoma (Solid 70%, Acinar 25%). (*A*) A 17 mm sized spiculated enhancing nodule is noted in left upper lobe. Activation map visually as heatmap, highlighting the most important region when it classified the given lesion as MPSol (micropapillary or solid pattern). All areas of the tumor were diffusely highlighted. (*B*) Surgical pathology demonstrates solid histologic pattern (marked with an asterisk) in most areas of the tumor that corresponds to activation map, Figure S8: A 58-year-old male with invasive lung adenocarcinoma (MP 85%, Acinar 10%). (*A*) A 22 mm sized irregular shaped enhancing nodule with internal cavitation is noted in right lower lobe. Activation map visually as heatmap, highlighting the most important region when it classified the given lesion as MPSol. All areas of the tumor were diffusely highlighted. (*B*) Surgical pathology demonstrates micropapillary histologic pattern (marked with an asterisk) in most areas of the tumor that corresponds to activation map, Table S1: Characteristics of Patients in the Training Set, Table S2: Characteristics of Patients in the Validation Set, Table S3: Cox Multivariate Regression Analysis of Progression Free Survival in Validation Set.
