# Supplementary material for: Deep Learning Analysis of CT Images Reveals High-Grade Pathological Features to Predict Survival in Lung Adenocarcinoma
Source: Cancers (Basel). 2021 Aug 13;13(16):4077. doi: 10.3390/cancers13164077 (PMC8391458; doi:10.3390/cancers13164077)
Supplement: Supplementary file 1 [file cancers-13-04077-s001.zip › cancers-1292240-supplementary.pdf]

Supplementary Data 1. Imaging Acquisition Parameters

CT images were obtained with the following parameters: (field of view, 30 to 36 cm; beam pitch, 1.35 or 1.375; gantry speed, 0.5 or 0.6 second per rotation; 120 kVp; 150–200 mA; and reconstruction interval, 1–2 mm). Scanning was performed 90 sec after contrast media administration (100 mL of Iomeron 300; Bracco, Milan, Italy) at a rate of 1.5 mL/sec. Scanning was performed from the thoracic inlet to the middle portion of the kidneys. All CT data were reconstructed using high-spatial-frequency and soft-tissue algorithms. Various CT scanners manufactured by different vendors were used, including 16-, 40-, and 64-MDCT scanners and a second-generation dual-source scanner as follows: a 64-MDCT scanner (Aquilion 64, Toshiba Medical Systems; LightSpeed VCT, GE Healthcare; Brilliance 64, Philips Healthcare), a 40-MDCT scanner (Brilliance 40, Philips Healthcare), and a 16-MDCT scanner (Somatom Sensation 16, Siemens Healthcare). To minimize variability due to image quality, only thin section images of less than 2mm were included in the analysis.

Table S1. Characteristics of Patients in the Training Set

Characteristic	No. of patients (n = 275) or No. of tumors (n = 290)	%
Age (mean ± SD) (yrs)	60 ± 9	
Sex		
Male	129	46.9
Female	146	53.1
Smoking		
Non-smoker	187	68.0
Ex-smoker	68	24.7
Current smoker	20	7.3
Pathologic invasive size	2.06 ± 1.03 cm	
T status		
pT1	215	78.2
pT2	54	19.6
pT3	6	2.2
N status		
pN0	247	89.8
pN1	10	3.6
pN2	18	6.6
Pathologic stage		
Ia	212	73.1
Ib	40	13.8
IIa	12	4.1
IIb	7	2.4
IIIa	19	6.6

Operation type		
Wedge resection	50	17.2
Segmentectomy	46	15.9
Lobectomy	194	66.9
Most predominant histologic pattern		
MPSol	28	9.7
Others	262	90.3
Second most predominant histologic pattern		
MPSol	34	11.7
Others	256	88.3

Abbreviations: SD, standard deviation.

Table S2. Characteristics of Patients in the Validation Set

	All		Definitive CCRT		Neoadjuvant CCRT		
**Characteristic**	**No. of patients (n = 416)**	**%**	**No. of patients (n = 416)**	**%**	**No. of patients (n = 416)**	**%**	**P value**
Age (mean ± SD) (yrs)	61 ± 10		61 ± 10		61 ± 9		0.377
Sex							
Male	270	64.9	133	67.2	137	62.8	
Female	146	35.1	65	32.8	81	37.2	
ECOG							0.302
0	68	16.4	28	14.1	40	18.3	
1	347	83.4	169	85.4	178	81.7	
2	1	0.2	1	0.5	0	0	
Smoking	224	53.7	103	52	120	55	0.603
T status (c or p)							0.003
T0	2	0.5	2	1	0	0	
T1	120	28.9	47	23.7	73	33.5	
T2	193	46.4	96	48.5	97	44.5	
T3	73	17.5	31	15.7	42	19.3	
T4	27	6.5	21	10.6	6	2.8	
Tx	1	0.2	1	0.5	0	0	
N status (c or p)							<0.001
N0	32	7.7	18	9.1	14	6.4	
N1	12	2.9	7	3.5	5	2.3	
N2	238	57.2	41	20.7	197	90.4	
N3	134	32.2	132	66.7	2	0.9	
TNM Stage							0.703
I	20	4.8	11	5.6	9	4.1	
II	11	2.6	6	3	5	2.3	
III	385	92.6	181	91.4	204	93.6	

Abbreviations: SD, standard deviation.

Table S3. Cox Multivariate Regression Analysis of Progression Free Survival in Validation Set

	**Univariable**		**Multivariable#**	
	**HR (95% CI)**	***p* Value**	**HR (95% CI)**	***p* Value**
Treatment: Neoadjuvant	1.212 (0.872-1.683)	0.253	-	-
Sex: male	1.754 (1.211-2.542)	**0.003***	1.410 (0.835-2.380)	0.199
Age	1.022 (1.004-1.040)	**0.018***	1.021 (1.003-1.040)	**0.022***
Smoking: yes	1.599 (1.143-2.236)	**0.006***		
ECOG (≥1)	0.944 (0.604-1.475)	0.800	-	-
MPSol prediction >0.5	1.258 (0.894-1.772)	0.188	1.178 (0.833-1.667)	0.354
TNM stage: I,II (Ref)				
TNM stage: III	1.008 (0.544-1.865)	0.981	-	-

*Bold face = $p < 0.05$, #Stratified by smoking
smoking status included both former and current smokers

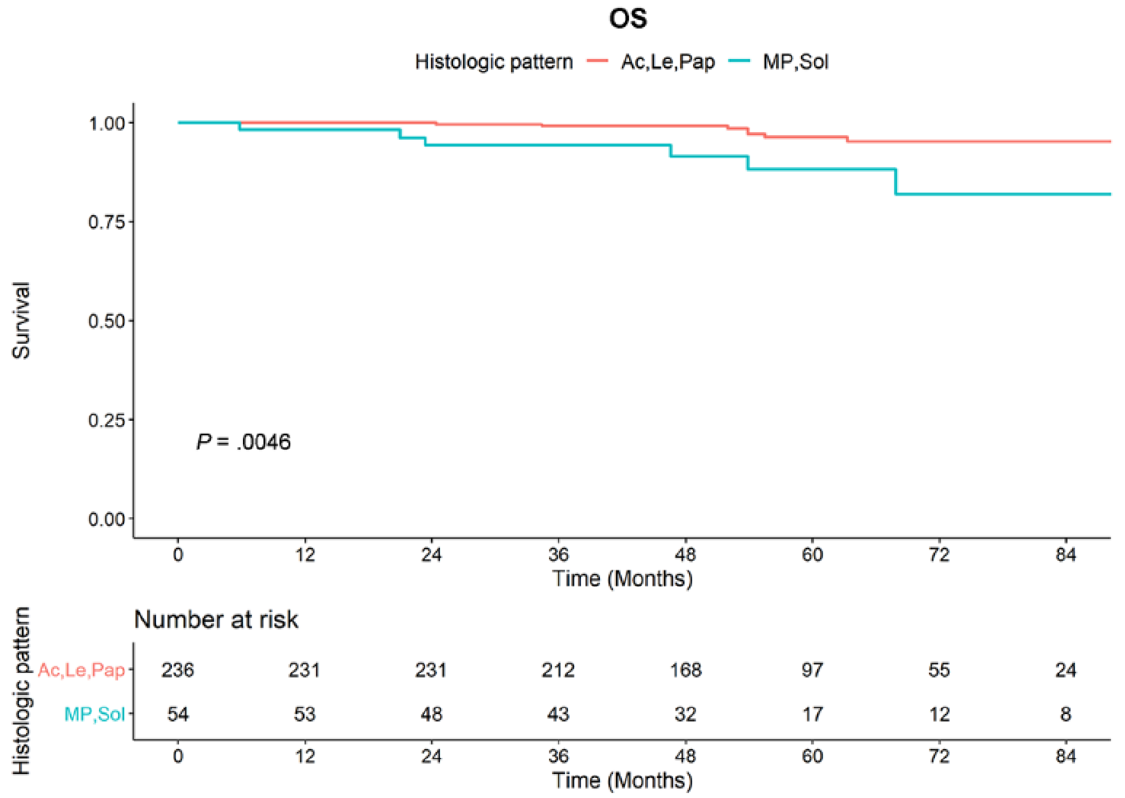

Figure S1. Survival curves for OS according to any high-grade histologic pattern of lung ADCs in training set. Overall survival was significantly different between lung ADCs with or without any high-grade histologic pattern such as micropapillary and solid pattern ($p = 0.005$, 5-year OS rate; 96.4% vs 88.2%).

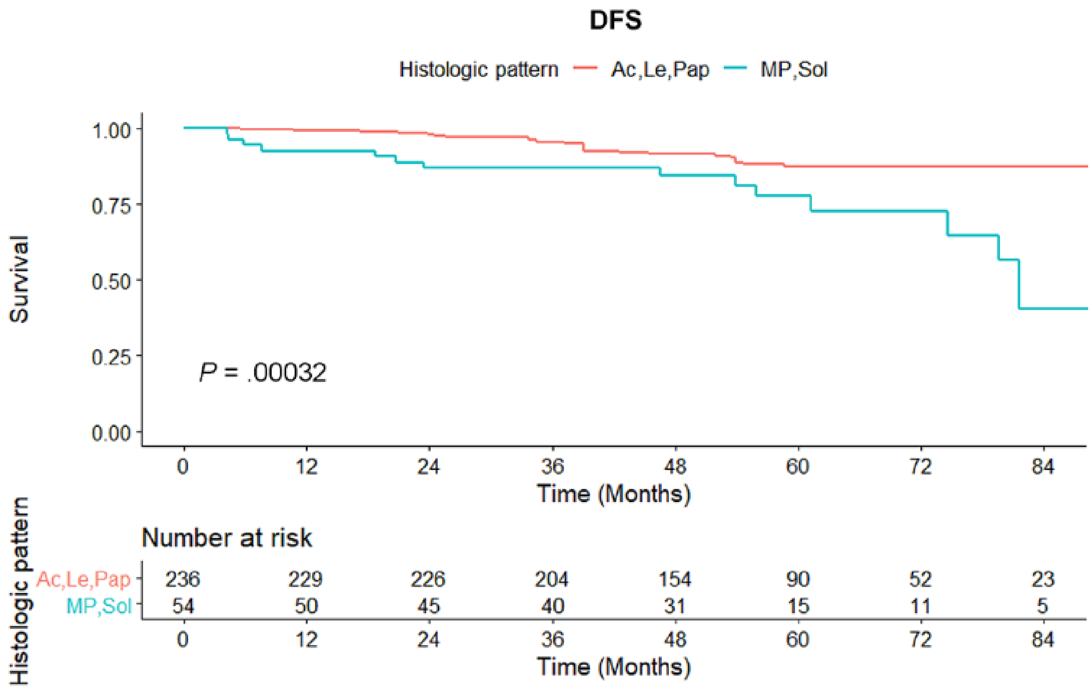

Figure S2. Survival curves for DFS according to any high-grade histologic pattern of lung ADCs in training set. DFS was significantly different between lung ADCs with or without any high-grade histologic pattern such as micropapillary and solid pattern ($p < 0.001$, 5-year DFS rate; 87.3% vs 77.8%).

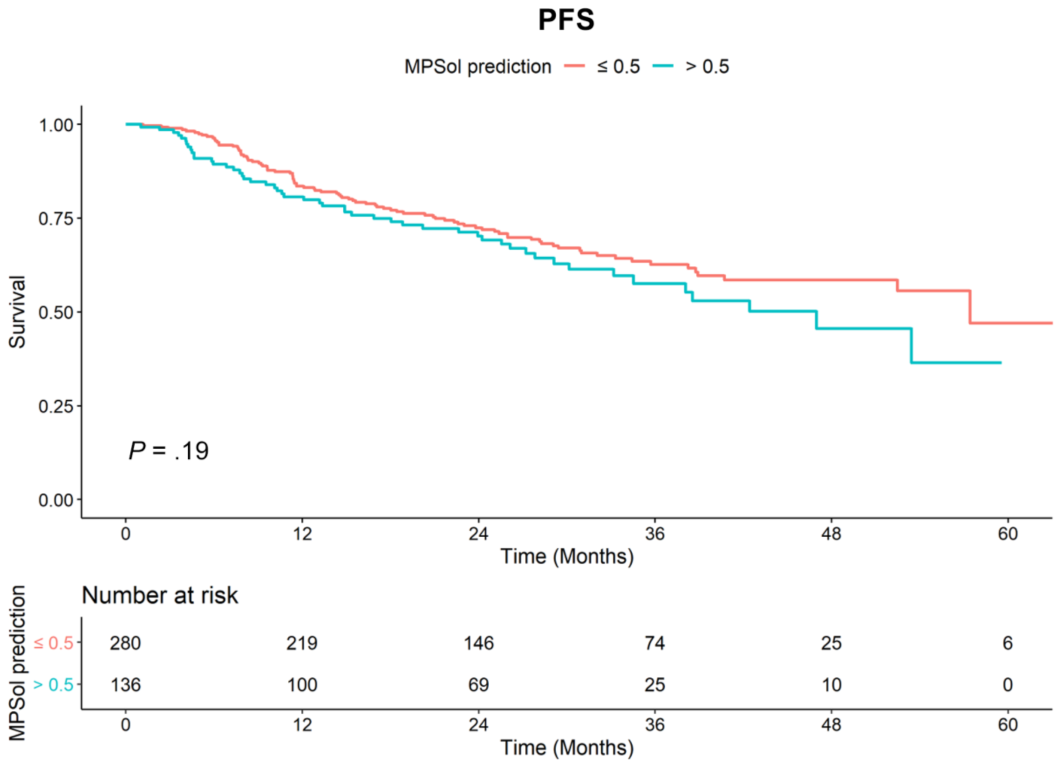

Figure S3. Survival curves for PFS according to MPSol probability estimated by MC3DN in the validation set. When the deep learning model was applied to the validation set, a high probability of a high-grade histologic pattern such as the micropapillary and solid pattern (MPSol) was associated with worse progression free survival (probability of MPSol > 0.5 vs < 0.5; 5-year PFS rate 47.0% vs 36.5%).

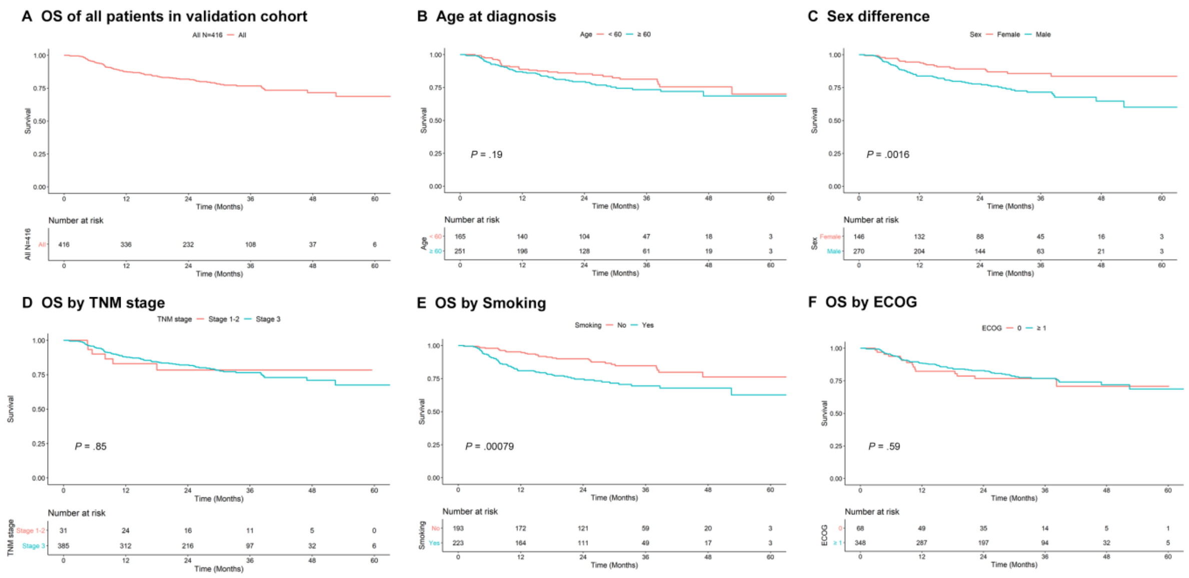

Figure S4. Survival curves for all patients in validation cohort and other clinical factors.

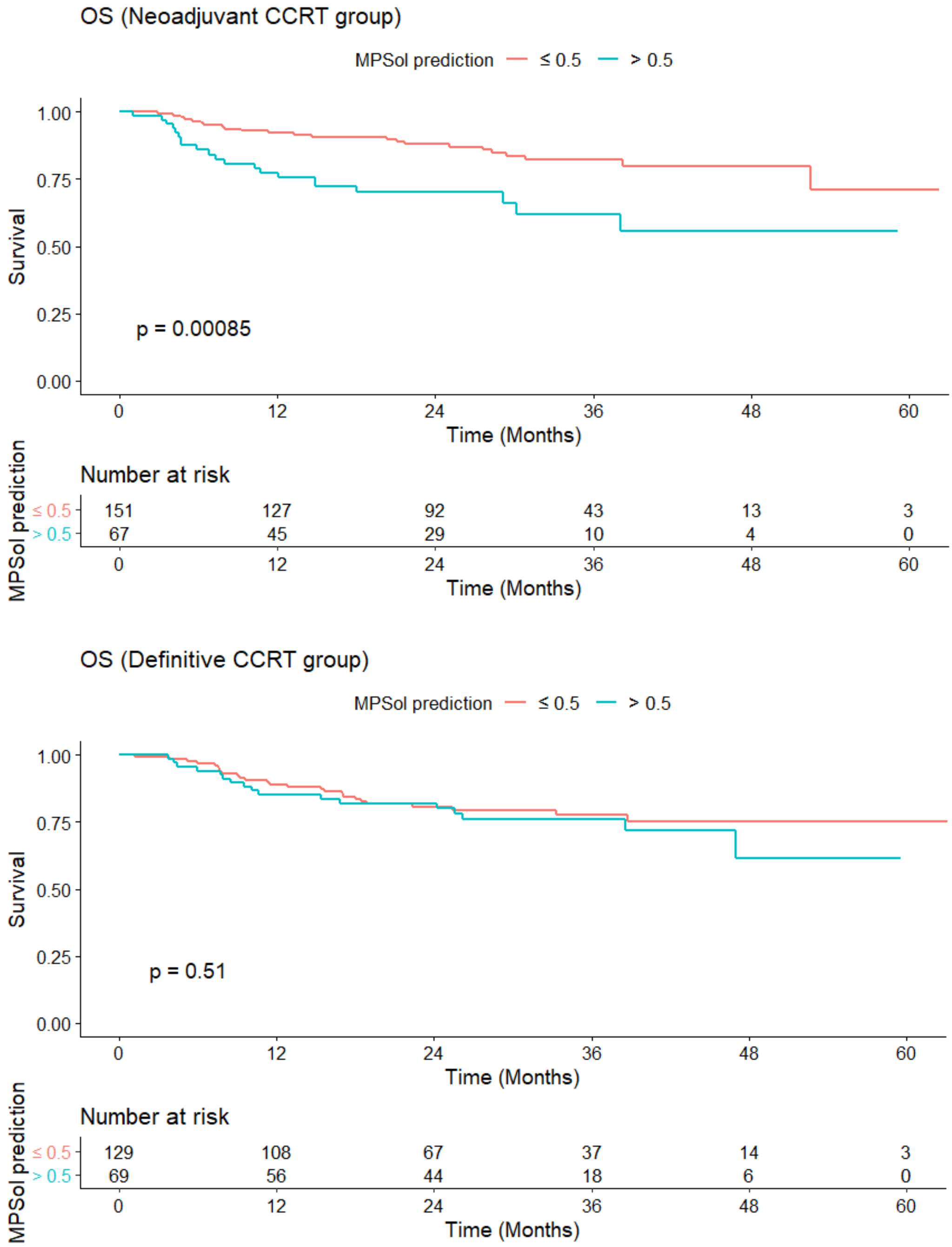

Figure S5. Survival curves for OS according to MPSol probability estimated by MC3DN in neoadjuvant or definitive CCRT group.

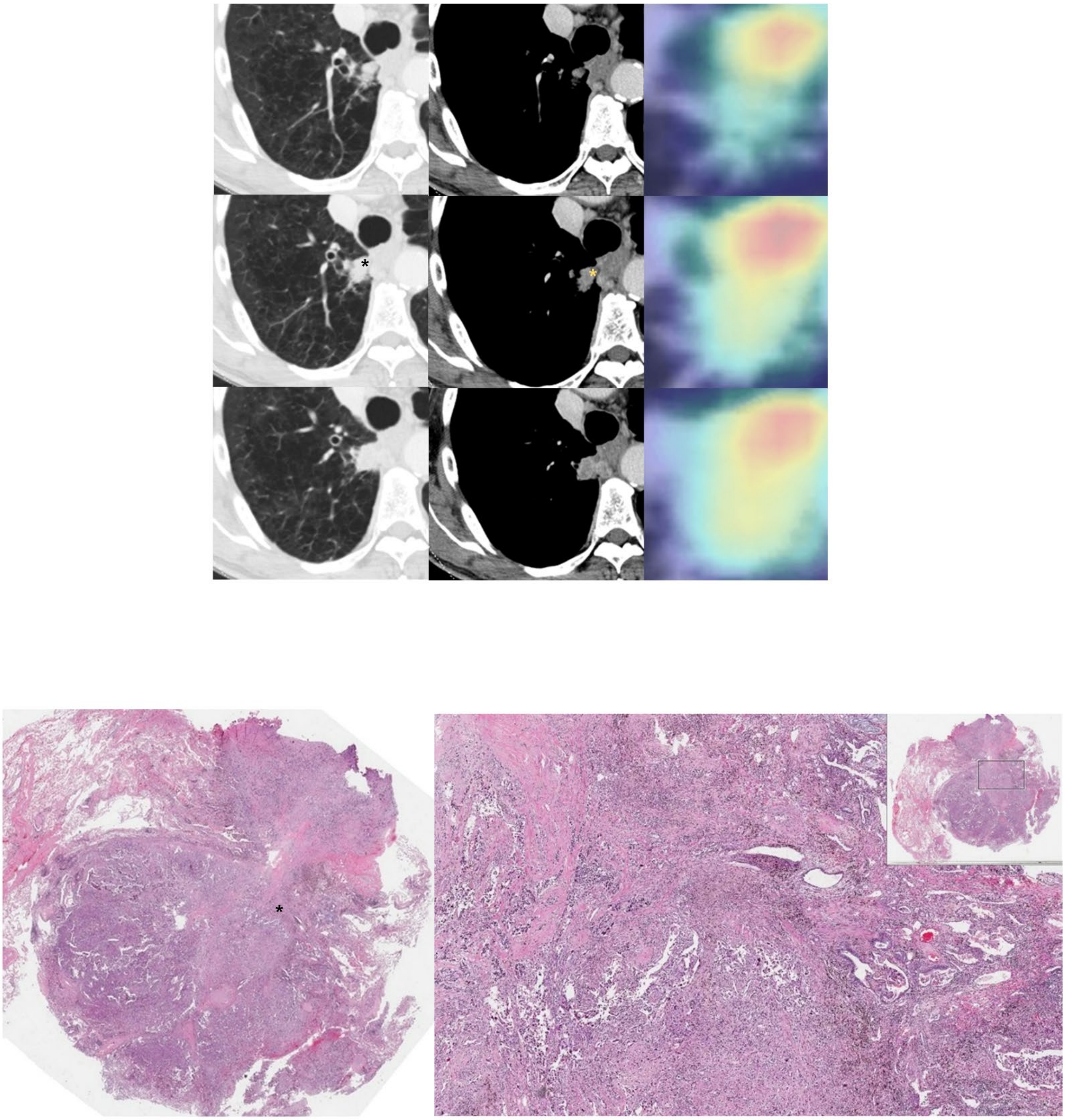

Figure S6. A 71-year-old male with invasive lung adenocarcinoma (Papillary 65%, Solid 30%). (*A*) A 30 mm sized lobulated heterogeneously enhancing mass is noted in right upper lobe. Activation map visually as heatmap, highlighting the most important region (marked with an asterisk) when it classified the given lesion as MPSol (micropapillary or solid pattern). A focal highlighted region is noted in central portion of

the tumor. (*B*) Surgical pathology demonstrates solid histologic pattern (marked with an asterisk) in central portion of the tumor which corresponds to activation map.

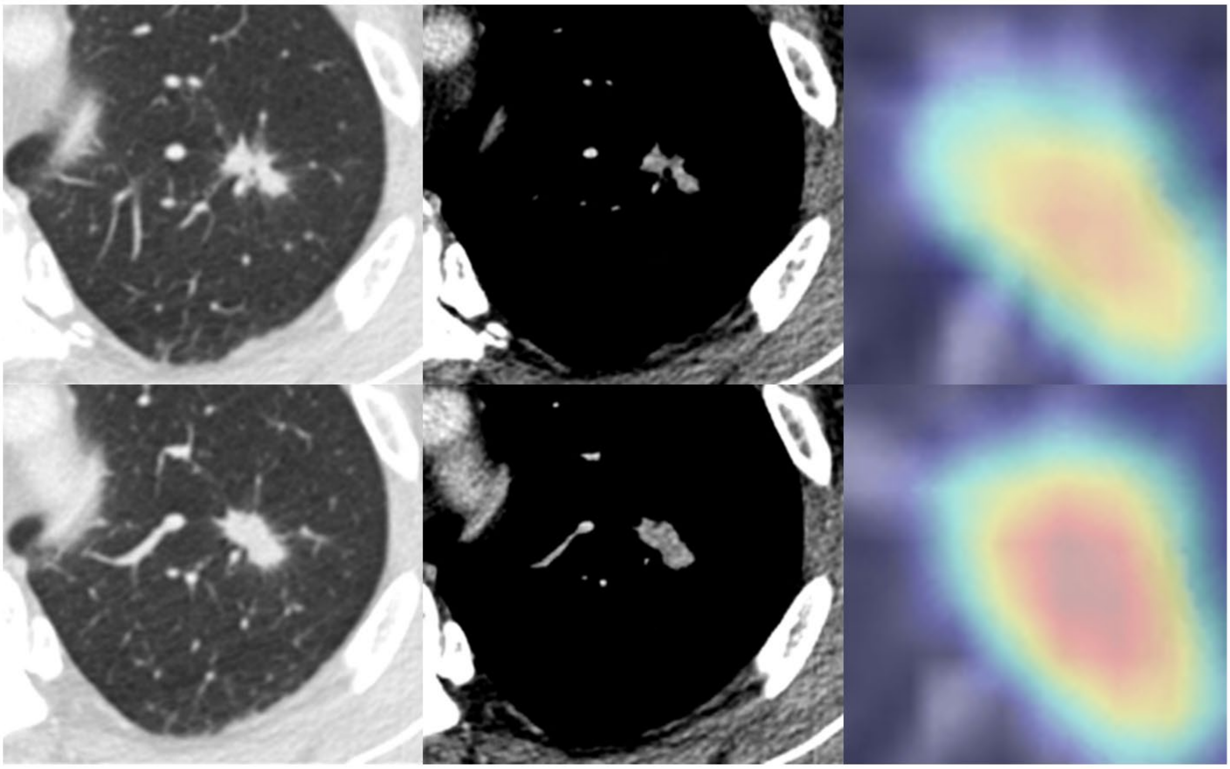

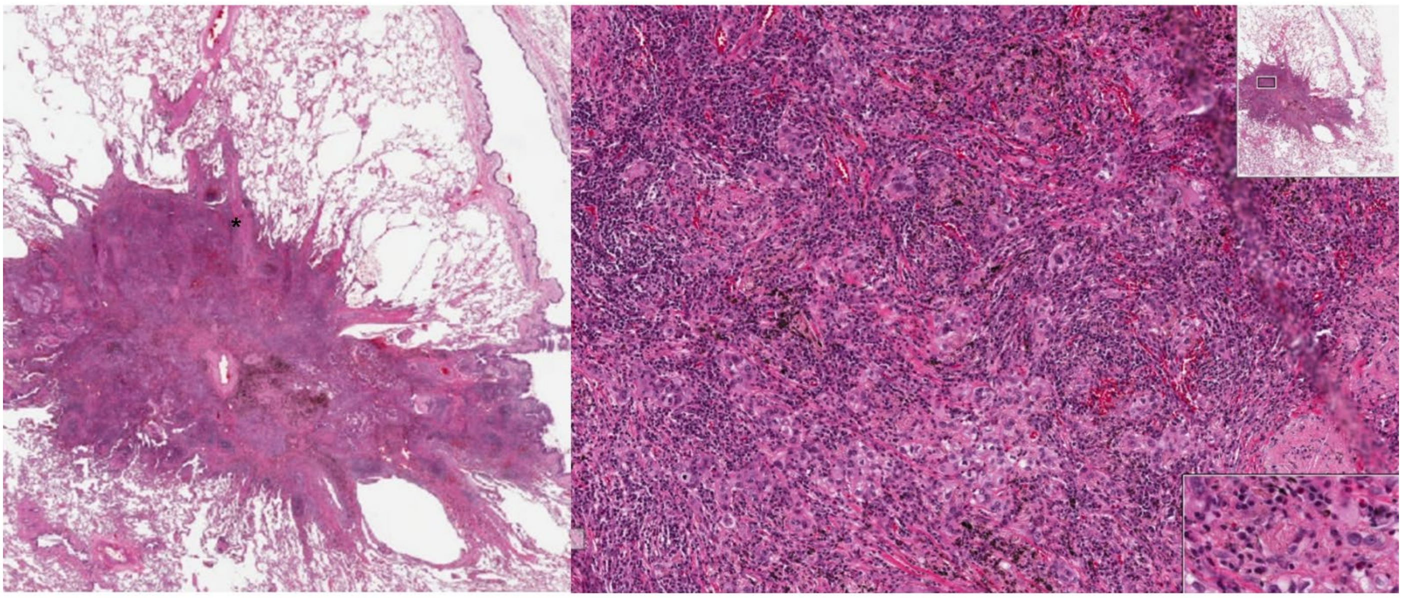

Figure S7. A 60-year-old male with invasive lung adenocarcinoma (Solid 70%, Acinar 25%). (*A*) A 17mm sized spiculated enhancing nodule is noted in left upper lobe. Activation map visually as heatmap, highlighting the most important region when it classified the given lesion as MPSol (micropapillary or solid pattern). All areas of the tumor were diffusely highlighted. (*B*) Surgical pathology demonstrates solid histologic pattern (marked with an asterisk) in most areas of the tumor that corresponds to activation map.

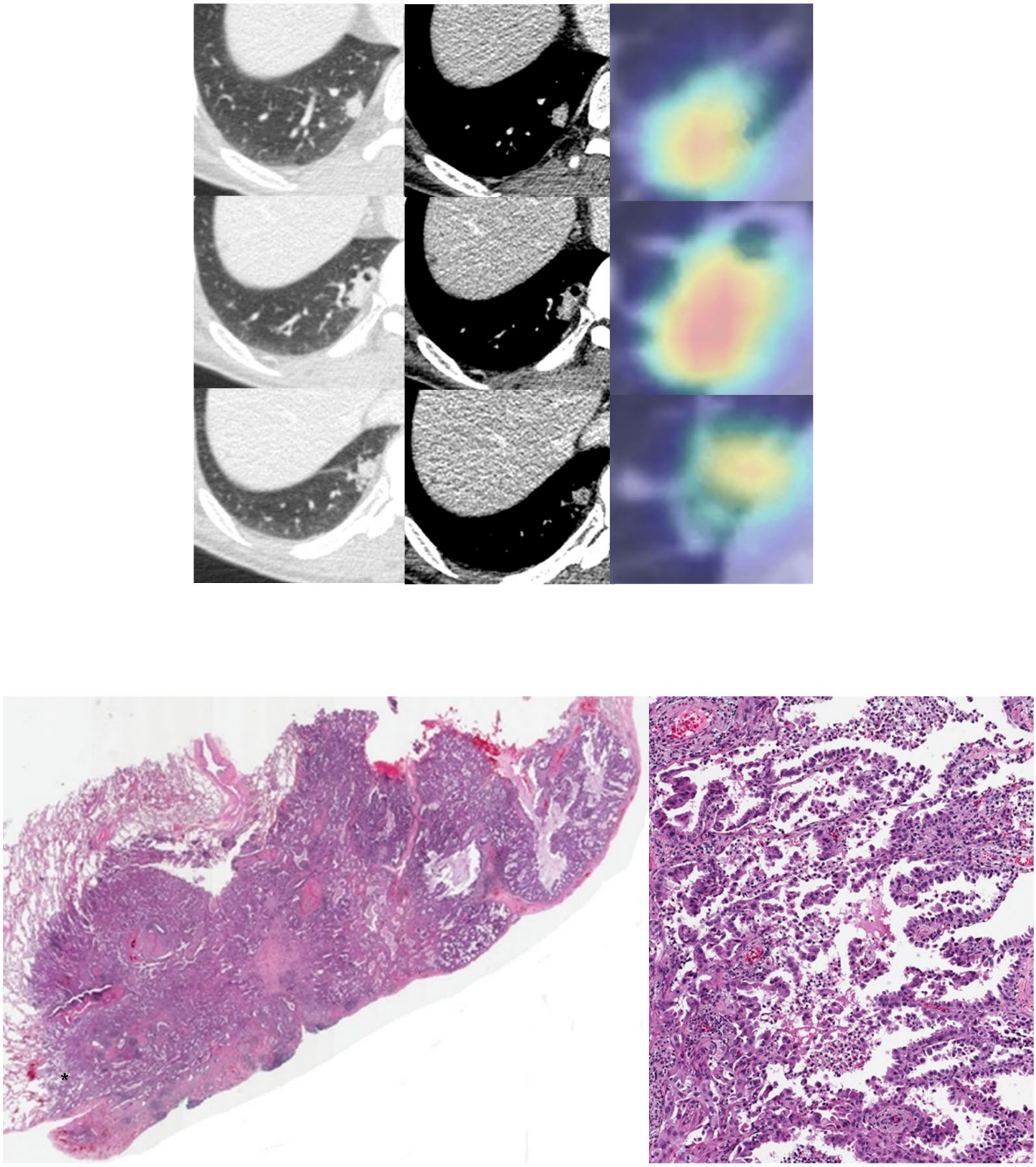

Figure S8. A 58-year-old male with invasive lung adenocarcinoma (MP 85%, Acinar 10%). (*A*) A 22mm sized irregular shaped enhancing nodule with internal cavitation is noted in right lower lobe. Activation map visually as heatmap, highlighting the most important region when it classified the given lesion as MPSol. All

areas of the tumor were diffusely highlighted. (*B*) Surgical pathology demonstrates micropapillary histologic pattern (marked with an asterisk) in most areas of the tumor that corresponds to activation map.
